# A Characteristic Back Support Structure in the Bisphenol A-Binding Pocket in the Human Nuclear Receptor ERRγ

**DOI:** 10.1371/journal.pone.0101252

**Published:** 2014-06-30

**Authors:** Xiaohui Liu, Ayami Matsushima, Miki Shimohigashi, Yasuyuki Shimohigashi

**Affiliations:** 1 Laboratory of Structure-Function Biochemistry, Department of Chemistry, Faculty of Sciences, and Risk Science Research Center, Kyushu University, Fukuoka, Japan; 2 Division of Biology, Faculty of Science, Fukuoka University, Fukuoka, Japan; Institut de Génomique Fonctionnelle de Lyon, France

## Abstract

The endocrine disruptor bisphenol A (BPA) affects various genes and hormones even at merely physiological levels. We recently demonstrated that BPA binds strongly to human nuclear receptor estrogen-related receptor (ERR) γ and that the phenol-A group of BPA is in a receptacle pocket with essential amino acid residues to provide structural support at the backside. This led BPA to bind to ERRγ in an induced-fit-type binding mode, for example, with a rotated motion of Val313 to support the Tyr326-binding site. A similar binding mechanism appears to occur at the binding site of the BPA phenol-B ring. X-ray crystal analysis of the ERRγ-ligand-binding domain/BPA complex suggested that the ERRγ receptor residues Leu342, Leu345, Asn346, and Ile349 function as intrinsic binding sites of the BPA phenol-B, whereas Leu265, Leu268, Ile310, Val313, Leu324, Tyr330, Lys430, Ala431, and His434 work as structural elements to assist these binding sites. In the present study, by evaluating the mutant receptors replaced by a series of amino acids, we demonstrated that a finely assembled structural network indeed exists around the two adjacent Leu^342^-Asn^346^ and Leu^345^-Ile^349^ ridges on the same α-helix 7 (H7), constructing a part of the binding pocket structure with back support residues for the BPA phenol-B ring. The results reveal that the double-layer binding sites, namely, the ordinary ligand binding sites and their back support residues, substantiate the strong binding of BPA to ERRγ. When ERRγ-Asn346 was replaced by the corresponding Gly and Tyr in ERRα and ERRβ, respectively, the binding affinity of BPA and even 4-hydroxytamxifen (4-OHT) is much reduced. Asn346 was found to be one of the residues that make ERRγ to be exclusive to BPA.

## Introduction

Bisphenol A (BPA) binds strongly to estrogen-related receptor γ (ERRγ), one of 48 human nuclear receptors [1], [2]. Although ERRγ, as well as its subtypes ERRα and ERRβ, is an orphan receptor whose physiological ligand is unknown even today [3], its transcriptional activity seems crucial, especially during differential gene expression and development [4], [5]. Also, it should be noted that one of the major physiological roles of the ERRs appears to control cellular energy metabolism [6].

The facts that ERRγ is rich in both the fetal brain and the placenta and that ERRγ is a probable candidate for involvement in prostatic growth and development seem to have important inferences for newborns [4], [7], [8]. Thus, the risk of exposure to BPA and its endocrine-disrupting activities has been a cause of concern, especially for fetuses, infants, and children, who are particularly vulnerable to the adverse effects of chemicals [9], [10]. So-called low-dose effects of BPA are increasingly being identified for many organ tissues and systems in mice and rats *in vivo* [11]–[16], and such effects would be magnified in infants and children. Research into the effects of binding between BPA and ERRγ has inevitably led to a hypothesis that the low dose effects of BPA are mediated through ERRγ and its specific target gene(s) [2], [17], [18].

ERRγ is constitutively active, exhibiting considerably high basal activity due to an activation conformation of the ligand-binding domain (LBD) with no ligand [1], [19]–[21]. Binding of BPA to ERRγ has almost no effect on the original active conformation [22], and thus almost all of the basal activity is preserved [1], [2]. ERRγ is a BPA-specific receptor, but the ERRγ-BPA complex behaves in the same manner as solo ERRγ unaccompanied with BPA. This makes the hypothesis mentioned above very difficult to test. Although BPA-binding has no detectable influence on the ERR receptor conformation, our recent studies have explored, for instance, the fact that the isopropyl-methyl group of Val313 rotates approximately 120° to sustain the tight interaction in the BPA/ERRγ-LBD complex and also to avoid a collision with the benzene A-ring of BPA [23]. Val313 is not a direct binding site of BPA, but a residue which assists or structurally supports the direct binding sites of BPA [24]. These results required us to inspect in detail all the BPA-binding sites of ERRγ.

**Figure 1:**
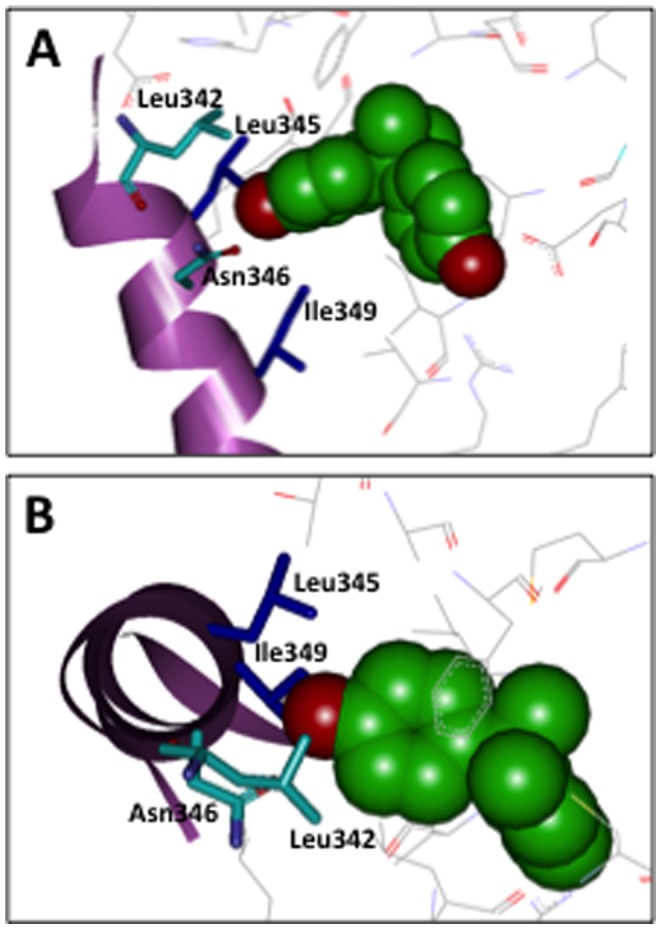
Characteristic 3D structural views of the bisphenol A-binding site in α-helix 7 (H7) of the ERRγ ligand-binding domain. (**A**) Side view of the interaction, and (**B**) top view of the interaction between bisphenol A and amino acid residues. (Protein Data Bank with accession code: 2E2R [22]).

On the central sp^3^ tetrahedral carbon atom (C*) of BPA, HO-C_6_H_4_-C*(CH_3_)_2_-C_6_H_4_-OH, there are two methyl groups and two phenol groups, A and B, respectively (Fig. 1). From the X-ray crystal structure of the BPA/ERRγ-LBD complex, a set of amino acid residues have been suggested to be in close proximity to the phenol-A group, and their roles as binding sites have been demonstrated by site-directed mutagenesis experiments [24]. Those include Glu275 (H3) and Arg316 (H5) for the phenol-hydroxyl group and Leu268, Leu271, Leu309, and Tyr326 for the phenol-A benzene ring, in addition to Ile279, Ile310, and Val313 for structural support of these residues.

When the amino acid residues of the ligand-binding pocket (LBP) of ERRγ were compared with those of ERRα and ERRβ, it was found that Asn346 is replaced by Gly and Tyr in ERRα and ERRβ, respectively. These replacements are the complete discrepancy seen in LBPs among all the ERR family (ERRα, β, and γ). The substitution of Asn to Gly in ERRα would expand the cavity of LBP and possibly allow much more flexible adaptation of ligands, but would perhaps be unfavourable for a tight binding of the BPA phenol-B group. On the other hand, the Asn→Tyr substitution in ERRβ apparently makes the cavity much narrower, which could prevent the binding of BPA to ERRβ.

In the present study, in order to better understand the characteristics of the binding of the BPA phenol-B ring to ERRγ, we carried out a series of site-directed point mutagenesis modifications. In our previous study, we demonstrated that there is a Leu268 in close proximity to the benzene B-ring of BPA, and this Leu268 is positioned like a clamp or double-hook to tightly connect the phenol-A and -B rings of BPA. In the present study, we first carefully checked the X-ray crystal structure of the ERRγ-LBD/BPA complex, including this Leu268, and especially with respect to the regions surrounding the BPA phenol-B ring. It was suggested that the Leu342, Leu345, Asn346, and Ile349 of ERRγ function as essential binding sites of the phenol-B group of BPA. It should be noted that direct binding site receptor residues are present on the same α-helix 7 (H7) of ERRγ-LBD (Fig. 1), forming the distinct adjacent ridges of Leu^342^-Asn^346^ and Leu^345^-Ile^349^. On the other hand, it was suggested that Leu265, Leu268, Ile310, Val313, Leu324, Tyr330, Lys430, Ala431 and His434 work as structural elements to assist these binding sites. In order to prove this suggestion, we evaluated a series of site-directed mutant receptors, and we carried out receptor-binding assays for expressed ERRγ-LBD mutant receptor proteins by using tritium-labelled BPA. In addition, the transcription activity of full-length mutant receptors was evaluated by a reporter gene assay using the HeLa cell line. The results indicated that Leu342, Leu345, Asn346, and Ile349 indeed play essential roles in capturing a single BPA molecule, and that the supportive roles of other surrounding residues are essential for this BPA binding. We here describe in detail the structure-activity studies on these amino acid residues that form imperative BPA-binding sites in ERRγ.

## Materials and Methods

### Chemicals

BPA and 4-α-cumylphenol were purchased from Tokyo Kasei Kogyo Co., Ltd. (Tokyo, Japan). 4-hydroxytamoxifen (4-OHT) was obtained from Sigma-Aldrich Inc. (St. Louis, MO, USA). [^3^H]BPA (8 Ci/mmol) was obtained from Moravek Biochemicals (Brea, CA, USA).

### Plasmid construction and site-directed mutagenesis

As previously reported [24], wild-type ERRγ-LBD encoding 222-458 residues was generated by PCR using a human kidney cDNA library (Clontech Laboratories, Mountain View, CA, USA) and cloned into the vector pGEX-6p-1 (GE Healthcare Life Sciences, Piscataway, NJ, USA) using the *Eco*RI and *Xho*I restriction enzyme sites. Full-length ERRγ was cloned into the vector pcDNA3.1(+) (Invitrogen, Carlsbad, CA, USA). The resulting plasmids were designated as pGEX-ERRγ-LBD and pcDNA3.1-ERRγ-Full, respectively.

A series of ERRγ mutants were prepared according to the manufacturer's instructions by using *PfuTurbo* DNA Polymerase (Stratagene, La Jolla, CA, USA) with pGEX-ERRγ-LBD or pcDNA3.1-ERRγ-Full as a template and a set of overlapping sense and antisense primer pairs. The mutations were introduced by PCR mutagenesis in a two-step reaction essentially as reported previously [24], [25]. Each mutant LBD or full-length ERRγ was amplified and cloned into the expression vector pGEX-6p-1 or pcDNA3.1(+) at the *Eco*RI and *Xho*I sites. The accuracy of all PCR product sequences was confirmed by using a CEQ™ 8800 Genetic Analysis System (Beckman Coulter, Fullerton, CA, USA).

### ERRγ-LBD protein expression

Wild-type and mutant ERRγ-LBD proteins were expressed as glutathione *S*-transferase (GST)-fused preparations in *E. coli* BL21 as described previously [1], [24], [25]. The receptor protein was purified first by affinity chromatography using a column (10×100 mm) of Glutathione-Sepharose 4B (GE Healthcare). After loading the protein solution, the column was incubated for 1 h at 4°C, and then washed three times with PBS containing 0.5% (v/v) Triton X-100 and once with sonication buffer (50 mM Tris-HCl (pH 8.0), 50 mM NaCl, 1 mM EDTA, and 1 mM dithiothreitol). The fusion protein was eluted with 50 mM Tris-HCl (pH 8.0) containing 20 mM glutathione (reduced form), which was removed by gel filtration on a column of Sephadex G-10 (15×100 mm; GE Healthcare) equilibrated with 50 mM Tris-HCl (pH 8.0). The purity was confirmed by SDS-PAGE using 12.5% polyacrylamide gel and stained by Coomassie brilliant blue. The protein concentrations were determined by the Bradford method [26].

### Circular dichroism (CD) spectra measurements

The GST-free ERRγ-LBD proteins were prepared from their GST-fused proteins for the purpose of CD spectra measurement only. GST was removed by using a specific enzyme PreScission Protease (GE Healthcare) on an affinity column of Glutathione-Sepharose 4B. After loading the solution of GST-ERRγ-LBD protein on the column, the resin was left to allow for incubation at 4°C for 4 h, and then eluted with a cleavage buffer of 50 mM Tris-HCl (pH 7.0) containing 150 mM NaCl, 1 mM EDTA, and 1 mM dithiothreitol. The eluate was diluted with the same buffer to obtain the solution of approximately 0.2 µM ERRγ-LBD protein for CD measurements. The concentration was determined by the Bradford protein method [26]. The Tris-HCl buffer used was judged relevant for CD measurements of proteins.

CD spectra were recorded at 5–95°C with 10°C intervals on a JASCO J-725 spectropolarimeter (JASCO Co., Tokyo, Japan) in a cell of 1 mm path length. Spectra were acquired over the 195–300 nm range at a scan rate of 5 nm/min. Four scans were accumulated to obtain a mean spectrum. The buffer solution itself depicted a rather intense absorption below 200 nm, and thus CD spectra of GST-free ERRγ-LBD proteins were normalized by subtracting the buffer scan recorded under the same conditions. The results were eventually analysed by the standard analysis software (JACSO) and expressed as the mean molar ellipticity [*θ*].

### Radio-ligand receptor-binding assays

#### Saturation binding

A saturation binding assay was conducted [27] using [^3^H]BPA. The reaction mixture was incubated at 4°C for 2 h with the receptor proteins—GST-fused wild-type ERRγ-LBD or its mutants—in 100 µl of binding buffer [10 mM HEPES (pH 7.5), 50 mM NaCl, 2 mM MgCl_2_, 1 mM EDTA, 2 mM CHAPS, and 2 mg/ml γ-globulin]. The assay was performed with or without the addition of unlabelled BPA (final concentration of 1.0×10^-5^ M) to quantify the specific and nonspecific binding. After incubation with 100 µl of 1% dextran-coated charcoal (DCC) (Sigma-Aldrich) [28] in PBS (pH 7.4) for 10 min at 4°C, the DCC-absorbed free radio-ligand was removed by the direct vacuum filtration method using a 96-well filtration plate (MultiScreen^HTS^ HV, 0.45 µm pore size; Millipore, Billerica, MA, USA) for the bound/free separation [27]. Radioactivity was determined on a liquid scintillation counter (LS6500; Beckman Coulter, Fullerton, CA, USA).

The data on the specific binding of [^3^H]BPA were first assessed by means of Scatchard plot analysis [29]. Then, these data were applied to a one-site binding hyperbola nonlinear regression analysis by the software package Prism (GraphPad Software Inc., La Jolla, CA, USA) to measure changes in the receptor density *B*_max_ and equilibrium dissociation constant *K*_d_. The saturation binding assay was performed at least three times.

#### Competitive binding

BPA was dissolved in a binding buffer containing 0.1% dimethyl sulfoxide (DMSO). Competitive binding assays were performed in the presence of GST-fused wild-type ERRγ-LBD or its mutants at the most appropriate concentration of each receptor. The reaction mixtures were incubated with [^3^H]BPA (final concentration 5 nM) at 4°C for 2 h, and the bound/free separation was carried out by the DCC method as described above. Radioactivity was determined on a liquid scintillation counter TopCount NXT (PerkinElmer Life Sciences Japan Co., Ltd., Tokyo, Japan). To estimate the binding affinity, the IC_50_ values (the concentrations for the half-maximal inhibition) were calculated from the dose-response curves evaluated by the nonlinear analysis program ALLFIT [30]. Each assay was performed at least three times.

### Cell culture and transient transfection assays

HeLa cells were maintained in Eagle's Modified Eagle Medium (EMEM) (Nissui, Tokyo, Japan) in the presence of 10% (v/v) fetal bovine serum at 37°C under 5% CO_2_. HeLa cells were first seeded for 24 h at 5×10^5^ cells/dish (6 cm in diameter), and then transfected with luciferase reporter plasmid pGL3/3×ERRE (3 µg), the expression plasmid of pcDNA3.1-ERRγ-Full (wild-type or mutant) (2 µg), and 10 ng/dish of pSEAP plasmid as an internal control in the medium (5 ml in total) with Plus reagent (10 µl; Invitrogen) and Lipofectamine LTX (15 µl; Invitrogen) according to the manufacturer's protocol. Approximately 24 h after this transfection, cells were harvested and plated onto 96-well plates at a concentration of 5×10^4^ cells per well.

Luciferase activity was measured after 24 h at 37°C under 5% CO_2_ by using a Luciferase Assay System (Promega, Madison, WI, USA) according to the manufacturer's instructions. Light emission was measured on a Wallac 1420 ARVOsx microplate reader (PerkinElmer). SEAP activity was assayed by using the Great EscAPe™ SEAP assay reagent (Clontech) according to the Fluorescent SEAP assay protocol [31], [32]. Secreted embryonic alkaline phosphatase (SEAP) is a reporter widely used to study promoter activity or gene expression. Cells treated with 1% BSA/PBS were used as a vehicle control. Values were computed as the fold inductions after normalization to SEAP activities. Each assay was performed in triplicate at least three times.

### Statistical analysis

Data are presented as the mean ± SD for indicated number of separate experiments. Statistical difference was determined by two-sided Student's t test. P-values less than 0.001 were considered significant.

### Computational structural analysis of ERRγ/bisphenol A complex

All molecular modeling studies were carried out using the molecular modeling software Discovery Studio 2.5 (Accelrys; San Diego, CA, USA). The protein structure of the ERRγ-LBD/BPA complex was downloaded from the RCSB Protein Data Bank (PDB) (http://www.pdb.org/pdb/home/home.do) (PDB accession code: 2E2R) and hydrogen atoms were added correctly. ERRγ-LBD/BPA complex was visualized and analyzed on the software. To identify amino acid residues constructed the LBP of ERRγ-LBD, the residues were carefully checked for their spatial interrelationships with BPA molecule, especially noting the interatomic distance and angle.

## Results and Discussion

### Essential amino acid residues as structural elements specific for BPA-binding

In order to successfully analyse the structure-activity relationship in proteins by replacing a series of amino acid residues with other amino acids, it is necessary to guarantee the purity of the protein expressed. In the present study, site-directed mutations were introduced into a series of amino acid residues on α-helix No. 7 (H7) of ERRγ. These included Leu342, Leu345, Asn346, and Ile349. Their back support residues such as Leu265, Leu324, Tyr330, Lys430, and His434 were also mutated. The mutations were carried out by the PCR mutagenesis method [24], [25]. For receptor-binding assays, mutant receptors were expressed as GST-fused ligand-binding domain (LBD) proteins in *E. coli* and purified by affinity-chromatography followed by gel-filtration. The purity of GST-fused ERRγ-LBD was examined by SDS-PAGE (12.5% polyacrylamide gel), and all of the GST-ERRγ-LBD mutants including the wild-type were judged to be sufficiently pure for use in the receptor-binding assays (Fig. S1). The purity of GST-free ERRγ-LBD was also guaranteed by SDS-PAGE (Fig. S1).

Another structural inspection that can be used to guarantee the quality of expressed proteins is analysis of the conformational uniformity and stability of ERRγ-LBD mutant receptors. This is particularly important for adequate evaluation and discussion of the structure-activity relationships between the wild-type and the mutant receptor proteins. In this study, the conformational quality of the expressed proteins was verified by the measurement of CD spectra. Because of the considerably large Cotton effects of GST, a protein with a mixed structure of α-helices and β-strands (Protein Data Bank accession code 2GSR) [33], we prepared GST-free ERRγ-LBD proteins for the purpose of CD spectra measurement only, as reported in a previous study [24]. Measuring the temperature-dependent CD spectra (5°C–95°C at 10°C intervals), it was demonstrated that all the proteins that were mutated at the LBP did indeed hold a properly folded conformation without any denaturation/misfolding at the temperature region 5°C–35°C (Fig. S2), which is the standard condition for temperature in the ordinary regular receptor binding assays. The thermal unfolding curves showed the approximately 55°C of the structural melting point (*T*m) for all of GST-free ERRγ-LBD proteins (Fig. S2).

### ERRγ position 346 requires Asn, but not Gly and Tyr, for specific BPA-binding

ERRα possesses Gly at position 402, which corresponds to Asn346 in ERRγ, and thus we replaced ERRγ-Asn346 by Gly. Although the resulting Asn346Gly-ERRγ mutant receptor exhibited sufficient specific binding (ca. 70% of the total binding) for [^3^H]BPA (Fig. S3), the equilibrium dissociation constant *K*_d_ value of [^3^H]BPA was 52.8 nM, approximately 11% of the binding affinity for the wild-type ERRγ (Table 1). This clearly indicates that the Asn→Gly substitution is greatly disadvantageous for the binding of BPA by ERRγ, probably due to the lack of hydrogen bonding between Asn346 and the phenol hydroxyl group of BPA. The results strongly suggest that it would be difficult to induce BPA to bind to ERRα.

**Table 1:** Receptor binding characteristics of [^3^H]BPA for wild-type ERRγ and its mutants in the saturation binding assay.

Amino acid residues of ERRγ receptors1)		Binding characteristics of [^3^H]BPA, *K*_d_ (nM)	Relative activity (%)	Receptor density for [^3^H]BPA, *B*_max_ (nmol/mg protein)
Position	Mutation			
Wild-type		5.70±0.88	100	18.40±0.78
Leu342	*Ala*	NSB2)	0	NSB
	*Val*	21.3±2.78	27	2.87±0.34
	*Ile*	14.1±3.31	40	2.75±0.50
Leu345	*Ala*	19.4±2.04	29	13.6±0.41
	*Ile*	58.1±8.75	10	4.07±0.21
	*Val*	57.3±8.08	10	7.72±0.55
Asn346	*Ala*	12.3±1.74	46	18.3±0.69
	*Gly*	52.8±6.63	11	11.8±0.57
	*Gln*	19.7±3.20	29	6.78±0.33
	*Tyr*	31.5±6.41	18	9.37±0.42
Ile349	*Ala*	15.9±0.99	36	5.92±0.12
	*Val*	6.15±1.46	93	8.84±0.86
	*Leu*	9.41±1.12	61	9.33±1.23
Leu265	*Ala*	NSB	0	NSB
Leu324	*Ala*	NSB	0	NSB
Tyr330	*Ala*	305±20.5	2	0.91±0.22
	*Phe*	11.7±1.75	49	7.37±0.33
Lys430	*Ala*	8.08±0.11	77	4.34±0.44
His434	*Ala*	NSB	0	NSB
	*Phe*	11.4±1.20	50	6.35±0.57

1) Specifically mutated residues are designated in italics. All the saturation binding assays to determine the dissociation constant (*K*_d_) and the receptor density (*B*_max_) for [^3^H]BPA were carried out at least three times.

2) NSB means "no specific binding" in the saturation binding assay.

**Figure 2:**
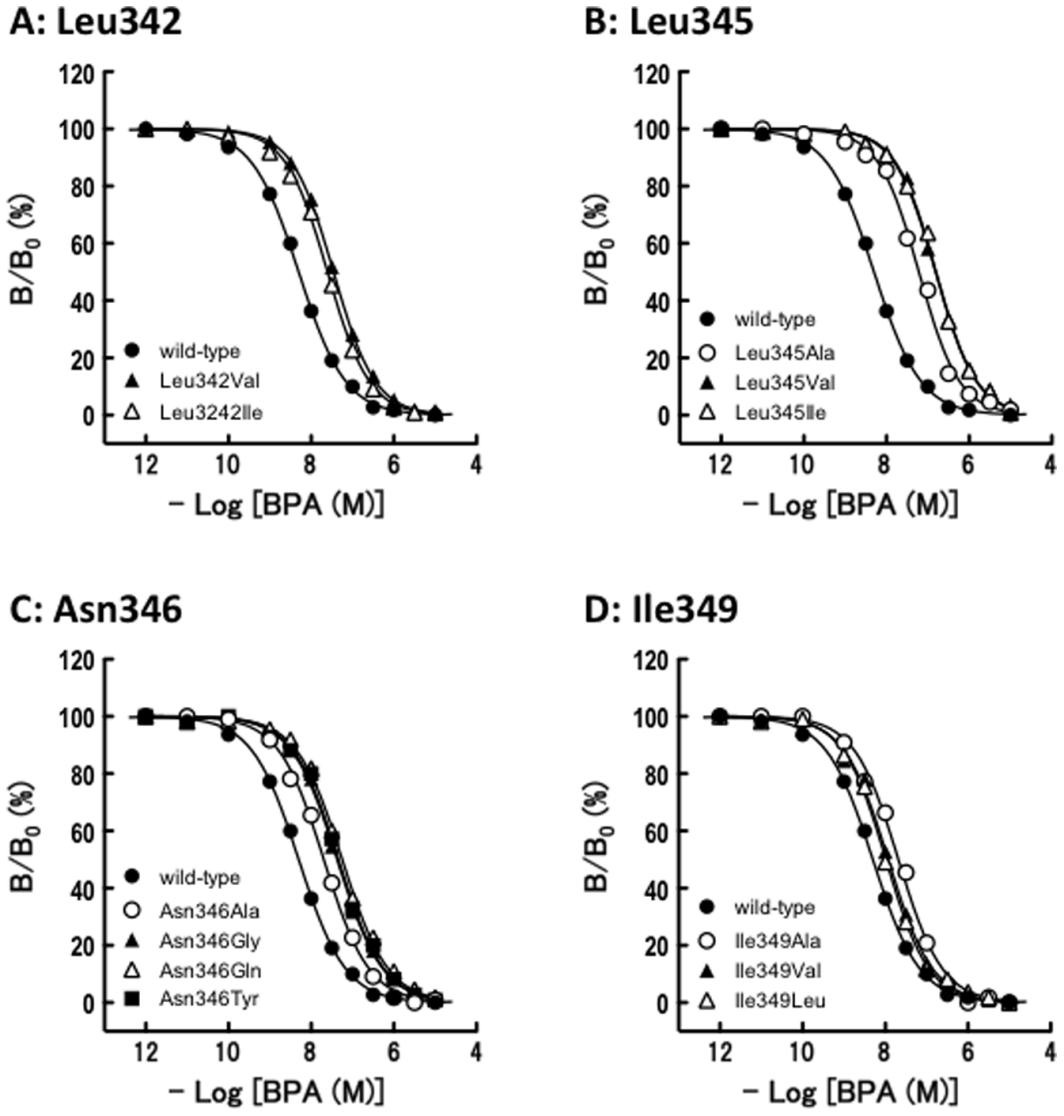
The homologous competitive binding assays between [^3^H]bisphenol A and non-labeled bisphenol A for the wild-type ERRγ-LBD and its mutants. The receptors used were the wild-type ERRγ and its mutant receptors. (**A**) Leu342-substituted ERRγ mutant receptors, (**B**) Leu345-substituted ERRγ mutant receptors, (**C**) Asn346-substituted ERRγ mutant receptors, and (**D**) Ile349-substituted ERRγ mutant receptors. The graphs show representative dose-dependent binding curves, which give the IC_50_ value closest to the mean IC_50_ from at least three independent experiments.

For the mutant receptors exhibiting a sufficient level of specific binding, the one-site binding hyperbola nonlinear regression analysis of saturation binding curves estimated the receptor density (*B*_max_: Table 1) for [^3^H]BPA (Fig. S3), in addition to *K*_d_ (Table 1). In the homologous competitive binding assay using tritium-labeled [^3^H]BPA and non-labeled BPA for the Asn346Gly-ERRγ mutant receptor, almost the same result as described above for [^3^H]BPA was obtained for non-labeled BPA (Fig. 2C). BPA was clearly weaker in this mutant receptor as compared with the wild-type ERRγ, the IC_50_ value being 42.4 nM [ca. 12% of the binding affinity (4.96 nM) for the wild-type ERRγ] (Table 2).

**Table 2:** Receptor binding potency of BPA, 4-α-cumylphenol, and 4-OHT in the competitive binding assay using [^3^H]BPA for human nuclear receptor ERRγ and its mutants with site-directed mutagenesis in the BPA binding site amino acid residues.

Amino acid residues of ERRγ receptors1)		Receptor binding potency IC_50_ (nM)		
Position	Mutation	BPA	4-α-cumylphenol	4-OHT
Wild-type		4.96±0.71	5.90±0.30	5.67±0.64
Leu342	*Ala*	*not carried out* 2)		
	*Val*	34.7±5.38	34.1±0.21	25.9±3.50
	*Ile*	24.5±4.53	23.7±0.99	16.0±2.87
Leu345	*Ala*	60.6±11.2	87.1±16.6	65.3±12.8
	*Val*	154±17.5	122±22.3	75.6±15.3
	*Ile*	158±19.8	138±29.8	79.0±11.5
Asn346	*Ala*	20.1±3.41	12.9±1.30	10.4±0.38
	*Gly*	42.4±5.44	26.2±4.40	26.9±2.03
	*Gln*	56.1±7.42	39.9±0.50	23.7±1.00
	*Tyr*	45.2±8.91	15.6±1.91	22.0±6.65
Ile349	*Ala*	20.1±3.24	19.8±2.20	12.7±3.23
	*Val*	11.7±1.50	12.9±0.33	12.5±2.29
	*Leu*	10.4±0.64	12.8±1.27	13.8±0.79

1) Specifically mutated residues are designated in italics.

2) Because there was "no specific binding" in the saturation binding assay, the competitive binding assay could not be carried out.

ERRβ has Tyr at position 321 corresponding to Asn346 in ERRγ. When this Tyr was placed at position 346 of ERRγ, the resulting Asn346Tyr-ERRγ exhibited a sufficient specific binding (ca. 60% of the total binding) for [^3^H]BPA (Fig. S3). The *K*_d_ value of [^3^H]BPA was 31.5 nM, approximately 18% of the binding affinity for the wild-type ERRγ (Table 1). In the competitive binding assay for this ERRγ mutant receptor using [^3^H]BPA as a tracer, non-labeled BPA exhibited an approximately 9-fold reduction in binding affinity (Table 2). All these results indicate that BPA binds strongly to ERRγ, but not to Asn346Tyr-ERRγ, indicating that the Asn→Tyr substitution at position 346 is disadvantageous for the acceptance of BPA. The structural conversion of the carboxyl amide CONH_2_ (Asn) into the phenol C_6_H_4_-OH group (Tyr) is clearly disadvantageous for the binding of BPA.

### Constructional importance of Tyr326 as a BPA-binding site

The *para*-hydroxyl group of Tyr at position 326 is in close proximity to the BPA phenol-B benzene ring. In the BPA/ERRγ-LBD binding complex analysed by X-ray crystallography, the distance between the *para*-hydroxyl-oxygen atom and benzene-carbon atoms was estimated to be 3.1–4.9 Å in the complex structure [22]. This spacing appeared to allow the intermolecular interaction to a sufficient degree. It should be noted, however, that the Tyr326→Phe mutant receptor lacking the *para*-hydroxyl group of Tyr-β-phenol was found to be as active as the wild-type [24], indicating that there is no interaction between Tyr326 and the BPA phenol-B benzene ring. This result was somehow surprising, since the two are in extremely close proximity.

The fact that the Phe326 mutant receptor is as active as the wild type with Tyr326 implies definite nonalignment or independence of the BPA benzene B-ring from the Tyr326-*para*-hydroxyl group. BPA and Tyr326 do not undergo an OH/π interaction, which is one of the strongest molecular interactions. Because the bond angle of C−O−H in Tyr-β-phenol is approximately 105°, the Tyr-β-phenol-*para*-hydroxyl hydrogen atom must be distant from the BPA benzene B-ring. This causes the Tyr326-β-phenol to act like a wall blocking the pocket from accepting the BPA phenol B-group. This is in stark contrast to the fact that the C2-C3 edge of the Tyr326-β-phenol ring is in close proximity (3.75 Å) to the benzene A-ring of BPA to undergo an *edge*-to-*face* π-π interaction, namely, the so-called CH/π interaction [24].

### The bisphenol A-binding site constructed by the Leu-Asn and Leu-Ile ridges

The binding site of the BPA phenol-B group in ERRγ is made up of Leu342, Leu345, Asn346, and Ile349, being at α-helix H7 of ERRγ-LBD (Fig. 1). Leu342 and Asn346 are on the same (*i*, *i*+4) ridge and the other Leu345 and Ile349 are on the adjacent (*i+3*, *i*+7) ridge in H7. Another important site is Tyr326 on the β-strand 1, and all these five receptor residues constrict a binding pocket for the BPA phenol-B group. In order to evaluate the contribution of H7 amino acid residues to the binding of BPA, Leu342, Leu345, Asn346, and Ile349 were mutated to Ala and some other structurally related amino acids.

#### Leu342

Leu342 was first replaced with Ala. The Leu342Ala-ERRγ mutant receptor exhibited no specific binding for [^3^H]BPA (Fig. S3) (Table 1), indicating that Leu342 is crucial for the binding of BPA to ERRγ. The Leu342 isobutyl side chain [-CH_2_CH(CH_3_)_2_] was thus clearly essential to hold the BPA's phenol-B benzene ring. When mutated to Val and Ile, the side chains of which are isopropyl [-CH(CH_3_)_2_] and *sec*-butyl [-CH(CH_3_)CH_2_CH_3_], respectively, BPA exhibited a 3–4 fold reduction in the binding affinity for these mutant receptors (Table 1). Similarly, in the homologous competitive binding assay of [^3^H]BPA/BPA for mutant receptors, BPA showed a 5–7 fold reduction in IC_50_ value (Fig. 2A) (Table 2). These results suggest that the Cβ-branched side chains are not suitable to accept the BPA molecule at position 342 of ERRγ.

#### Leu345

In our previous study, we demonstrated that, when BPA binds to ERRγ Leu345 makes a back-and-forth rotation of its side chain isopropyl group to adopt a BPA molecule [23]. A strong hydrophobic interaction between the phenol-B benzene ring of BPA and the isopropyl group of Leu345 was eventually demonstrated. The Leu345→Ala substitution, namely, the elimination of the isopropyl group from the Leu side chain β-methylene, resulted in a drop (approximately 3.5-fold) in the receptor affinity of [^3^H]BPA (Table 1). When Leu345 was replaced with Val, the resulting mutant receptor exhibited a sharp drop in its affinity for [^3^H]BPA; *i.e*., an almost 10-fold decrease in the affinity for [^3^H]BPA in the saturation binding assay (Table 1) and an approximately 30-fold decrease in the affinity for [^3^H]BPA in the [^3^H]BPA/BPA homologous competitive receptor binding assay (Fig. 2B) (Table 2).

Although the isobutyl group of the Leu side chain could potentially make a back-and-forth rotation, the isopropyl group of the Val side chain cannot make such a rotation. Since Val's isopropyl side chain can rotate freely around the Cα-Cβ axis, it would stay at the most stable location. It is highly likely that this location resulted in a sterical hindrance just due to its unfavourable rotatory χ angle around the Cα-Cβ axis, causing a drastic drop in the receptor affinity of BPA. A similar phenomenon was observed for the Leu345Ile mutant receptor, which showed an approximately 30-fold decrease in the binding of BPA in the homologous competitive binding receptor assay (Fig. 2B) (Table 2). Ile's side chain *sec*-butyl group also cannot rotate in a back-and-forth manner. All these results indicate that Leu345 is one of the intrinsic determining factors of BPA's highly specific binding ability for ERRγ.

#### Asn346

The results of the mutation of Asn346 to Gly and Tyr are described above. In addition, in order to evaluate the structural importance of this Asn346 residue, it was also mutated to Ala. The resulting Asn346Ala-ERRγ mutant receptor exhibited a fairly high specific binding (ca. 90% of the total binding) for [^3^H]BPA (Fig. S3). However, this well-folded Asn346Ala-ERRγ-LBD protein exhibited a distinctly reduced (2–4 times) binding affinity of BPA (Tables 1 and 2) (Fig. 2C), implying that Asn346's side chain carboxamide methyl group is engaged in the hydrogen bond with the hydroxyl group in the BPA's phenol-B. Gln is a homolog of Asn, and the difference in their side chains is one methylene chain in length; *i.e*., -CH_2_CONH_2_ for Asn and -CH_2_CH_2_CONH_2_ for Gln. Since the Asn346Gln-ERRγ mutant receptor exhibited a 3-10 fold reduction in the affinity for BPA (Tables 1 and 2), this Asn346↔BPA hydrogen bond was judged to be highly specific and effective.

#### Ile349

When Ile349 was replaced with Ala, the resulting Ile349Ala-ERRγ exhibited a 3-4 fold reduction in affinity for [^3^H]BPA and BPA (Tables 1 and 2). This indicates the importance of the *sec*-butyl side chain for Ile to bind a BPA molecule. When Ile349 was replaced with Val and Leu, the activity decrements were rather small: 8-65% for [^3^H]BPA (Table 1) and 110-135% for BPA (Fig. 2D) (Table 2). However, the importance of Ile349 is beyond doubt, since each of the Ile349Ala-ERRγ, Ile349Val-ERRγ, and Ile349Leu-ERRγ mutant receptors showed clearly lower *B*_max_ values (Table 1).

Collectively, the amino acid residues of Leu342, Leu345, Asn346, and Ile349, which are present at the α-helix H7 of ERRγ, were judged to be authentic binding sites of BPA. Although their interactions with BPA molecules vary considerably, their significance is absolute and fundamental, as evidenced by the site-directed mutagenesis experiments.

### Amino acid residues that support the binding sites of BPA's phenol-B

**Figure 3:**
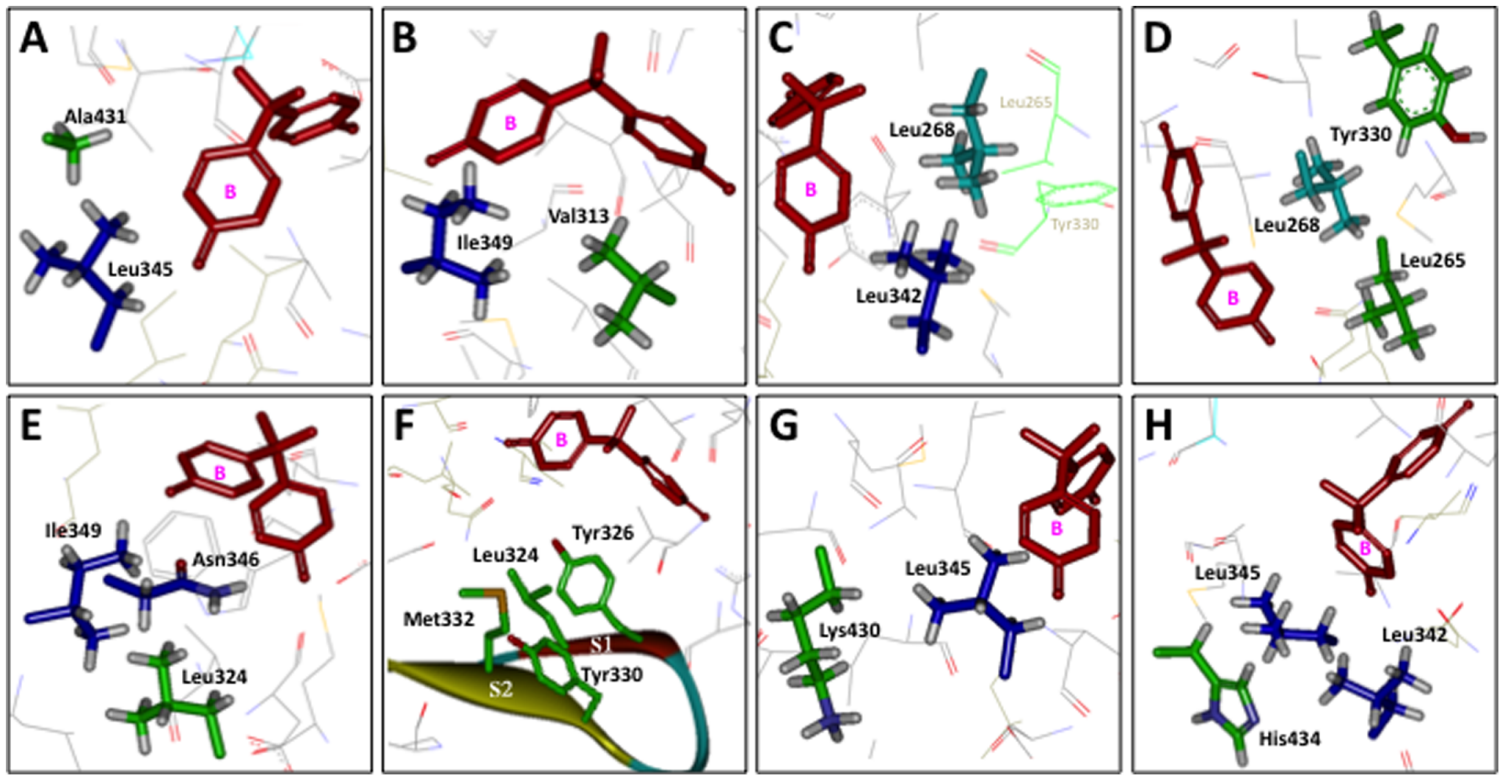
Structural interrelationships between the bisphenol A-binding site amino acid residues Leu^342^-Asn^346^/Leu^345^-Ile^349^ and their back support residues present in the binding pocket of ERRγ-LBD. (**A**) Leu345 and its back support residue Ala431. There is no direct contact between Ala431 and BPA. (**B**) Ile349 and its back support residue Val313. There is no direct contact between Val313 and BPA. (**C**) Leu342 and its back support residue Leu268. (**D**) Leu268 and its back support residues Leu265 and Tyr330. This Leu268 binds directly to the phenol-A and B rings of BPA as a double-hook to connect both tightly. (**E**) Ile349/Asn346 and their simultaneous back support residue Leu324. (**F**) Four amino acids, Leu324, Tyr326, Tyr330, and Met332, stand on the pleated β-sheet, which makes a bottom plate of ERRγ-LBP. (**G**) Leu345 and its back support residue Lys430. (**H**) Leu342/Leu345 and their simultaneous back support residue His434.

In our previous study, several amino acid residues were found to assist or structurally support the binding site amino acid residues, the side-chains of which participate in formation of a receptacle pocket for the BPA's phenol-A group [24]. Those included Ile279, Ile310, and Val313. In the present work, we also attempted to explore the surroundings of the amino acid residues of Leu342, Leu345, Asn346, and Ile349, which have been identified as the binding sites of BPA's phenol-B group. Ala431, which is located within 4.5 Å of the BPA's sp^3^ methyl group, was found to be in close proximity to Leu345 (Fig. 3A), and was very likely to provide structural support for the binding site.

In the present study, Val313 was again identified to be important as a structurally supporting residue. When the region surrounding Ile349 was checked carefully, Val313 was found to make an important contribution to the structurally supportive environment (Fig. 3B). In fact, the Ala-substitution of Val313 resulted in a considerable reduction (approximately 5-fold) in the binding affinities of BPA (Table 3). It should be noted that, when the structure of ERRγ-LBD with BPA was compared with the structure of ERRγ-LBD without BPA, the isopropyl-methyl group of Val313 was rotated about 120° to prevent the collision with the BPA's phenol-A benzene ring [24]. Val313 was thus shown to be one of the key residues for construction of the BPA-binding site in ERRγ-LBD, serving to hold the BPA-binding site amino acid residues in the proper positions. It is of note that there is no direct contact between Val313 and BPA.

**Table 3:** Receptor binding potency of BPA, 4-α-cumylphenol, and 4-OHT in the competitive binding assay using [^3^H]BPA for human nuclear receptor ERRγ and its mutants with site-directed mutagenesis in the back support residues of BPA binding sites.

Amino acid residues of ERRg receptors1)		Receptor binding potency IC_50_ (nM)		
Position	Mutation	BPA	4-α-cumylphenol	4-OHT
Wild-type		5.64±0.17	5.72±0.56	5.67±0.64
Leu265	*Ala*	*not carried out* 2)		
Leu2683)	*Ala*	*not carried out*		
Ile3103)	*Ala*	21.9±0.99	19.5±2.23	20.2±2.33
Val3133)	*Ala*	26.8±2.12	21.7±1.88	32.5±6.36
	*Phe*	*not carried out*		
	*Leu*	16.0±1.41	13.5±1.55	25.3±1.74
	*Ile*	*not carried out*		
Leu324	*Ala*	*not carried out*		
Tyr330	*Ala*	361±39.3	422±12.0	617±12.5
	*Phe*	16.1±0.58	9.32±0.49	9.33±0.90
Lys430	*Ala*	9.70±0.37	9.06±0.58	4.10±0.28
His434	*Ala*	*not carried out*		
	*Phe*	13.7±0.66	5.12±0.26	234±17.5

1) Specifically mutated residues are designated in italics.

2) Because there was "no specific binding" in the saturation binding assay, the competitive binding assay could not be carried out.

3) The results of ERRγ mutant receptors of Leu268, Ile310, and Val313 for BPA, except for those for 4-OHT and 4-α-cumylphenol, were re-collected from the previous report [24] for providing easiness in understanding.

Ile310 was found to also be one of the amino acids in the region surrounding Ile349, whereas Leu268 was one of amino acids in the region surrounding Leu342. As reported previously [24], Leu268 is a phenol-B binding site of BPA and is positioned like a clamp or double-hook to tightly connect both of BPA's phenol-benzene rings. Leu268 was found to be in close proximity to Leu342 (Fig. 3C). In this study, we further found that Leu265 and Tyr330 were back support residues of Leu268 (Fig. 3D). As a result, Leu268 serves both as a direct BPA-binding site and a back support residue, and Leu265 and Tyr330 serves only as a back support residue. Other newly speculated possible back support residues include Leu324 for Asp346 and Ile349, His434 for Leu342 and Leu345, and Lys430 for Leu345 (Fig. 3). Thus, Leu265, Leu324, Tyr330, Lys430, and His434 were mutated to Ala in order to determine their structural importance in the construction of BPA-binding pockets.

#### Leu265

Leu265 is present in the H3 α-helix, and we speculated that this Leu265 was one of the back support residues of Leu268 (Fig. 3C). As seen for the Leu342Ala-ERRγ mutant receptor, Leu265Ala-ERRγ exhibited no specific binding for [^3^H]BPA (Table 1), indicating that Leu265 is critical for the binding to BPA. We could not perform both the saturation and competitive binding assays for [^3^H]BPA. Since there is no direct contact between Leu265 and BPA, this Leu265 residue is judged to be a genuine back support residue of Leu268.

#### Leu324

Leu324 is on the S1 β-strand together with Tyr326. The S1 and S2 β-strands form a pleated sheet, which corresponds to a bottom plate of the ERRγ ligand-binding pocket. Leu324 was speculated to be a back support residue of both Asn346 and Ile349 (Fig. 3E), and Leu324Ala-ERRγ exhibited no specific binding for [^3^H]BPA (Table 1). These results clearly imply that Leu324 is crucial for ERRγ to bind BPA, particularly as an underlying support of Asn346 and Ile349.

#### Tyr330

Tyr330 is on the S2 β-strand, which forms a pleated sheet together with the S1 β-strand. The resulting pleated β-sheet, a bottom plate of the ERRγ-LBP, has four amino acids (Leu324, Tyr326, Tyr330, and Met332) which stand close together inside the pocket (Fig. 3F). In addition to Leu265, we speculated that Tyr330 was a possible back support residue of Leu268 (Fig. 3C). When [^3^H]BPA was examined in a saturation binding assay with the Tyr330Ala-ERRγ mutant receptor, [^3^H]BPA showed a more than 50-fold reduction in *K*_d_ value (Table 1). In the homologous competitive binding assay of [^3^H]BPA vs. BPA, BPA showed a more than 60-fold reduction in IC_50_ value (Table 3). All these data indicate that Tyr330 is crucial as an underlying support of Leu268.

The importance of the Tyr-phenol hydroxyl group was proved by examination of the Tyr330Phe-ERRγ mutant receptor. In the saturation binding assay, [^3^H]BPA showed an approximately 2-fold reduction in *K*_d_ value (Table 1), and in the homologous competitive binding assay of [^3^H]BPA/BPA, BPA showed an approximately 3-fold reduction in IC_50_ value (Table 3). These results imply that the *para*-hydroxyl group of Tyr330 is an important structural element for the construction of ERRγ-LBP on the bottom pleated β-sheet.

#### Lys430

Lys430 is present in the H11 α-helix, and we speculated that this Lys430 was a back support residue of Leu345 (Fig. 3G). When the Lys430Ala-ERRγ mutant receptor was examined, [^3^H]BPA showed only a 40% reduction in *K*_d_ value in the saturation binding assay (Table 1), and a 70% reduction in IC_50_ value in the homologous competitive binding assay of [^3^H]BPA/BPA (Table 3). These results suggest that Lys430's back supporting ability is rather weak as compared to other residues. However, as shown in Table 1, the significance of Lys430 was evidenced by the much lower *B*_max_ value of Lys430Ala-ERRγ. These findings suggested that Lys430 is important for the construction of a sound and complete ERRγ-LBP.

When 4-hydroxytamoxifen (4-OHT) was tested in a heterogeneous binding assay using [^3^H]BPA for Lys430Ala-ERRγ, it showed a rather stronger receptor binding potency (IC_50_ = 4.10 nM) as compared with the potency (5.67 nM) for the wild-type ERRγ (Table 3). This reinforced (about 40%) binding potency appeared to be due to the structural liberation of Leu345 from Lys430. Ala at position 430 might have been a better binding site for 4-OHT.

#### His434

His434 is present also in the H11 α-helix, and we speculated that this His434 was a back support residue of Leu342 and Leu345 (Fig. 3H). It was first replaced with Ala, and the resulting His434Ala-ERRγ mutant receptor exhibited no specific binding for [^3^H]BPA (Table 1). These results clearly imply that His434 is crucial for the binding of BPA by ERRγ, particularly as a primary support of Leu342 and Leu345. When His was replaced with another aromatic amino acid, Phe, the resulting His434Phe-ERRγ mutant receptor exhibited considerably reduced activities of BPA. When His434Phe-ERRγ was examined, [^3^H]BPA showed a 100% reduced *K*_d_ value in the saturation binding assay (Table 1), and a 140% reduced IC_50_ value in the homologous competitive binding assay of [^3^H]BPA/BPA (Table 3). These results indicate that His434 makes an indispensable contribution to the back support, and thus Phe cannot replace it. The significance of His434 was further shown by the much lower *B*_max_ value of His434Phe-ERRγ (Table 1).

When 4-α-cumylphenol was tested in the heterogeneous binding assay using [^3^H]BPA for His434Phe-ERRγ, this compound exhibited an almost unchanged high receptor binding potency (IC_50_ = 5.12 nM), just like the potency (5.72 nM) for the wild-type ERRγ (Table 3). This appeared to be due to the structural role of His434 as a primary support of Leu342 and Leu345, and perhaps Phe placed at position 434 provided better back support for these Leu residues, allowing them to accept the phenyl-B benzene ring of 4-α-cumylphenol. Interestingly, the opposite results were obtained for 4-OHT. 4-OHT exhibited a more than 40-fold reduction in receptor-binding potency (IC_50_ = 234 nM) for His434Phe-ERRγ as compared with that (5.67 nM) for the wild-type ERRγ (Table 3). It is clear that Phe434 is quite unfavourable for the binding to 4-OHT, but not for the binding to 4-α-cumylphenol.

### Binding of 4-α-cumylphenol and 4-hydroxytamoxifen

In this study we demonstrated that one of the two phenol-hydroxyl groups of BPA makes a hydrogen bond with Asn346. The other phenol-hydroxyl group simultaneously forms hydrogen bonds with Glu275 and Arg316 [24], [25]. 4-α-Cumylphenol, HO-C_6_H_4_-C(CH_3_)_2_-C_6_H_5_, lacks one of the two phenol-hydroxyl groups of BPA, and it was found to bind to ERRγ as potently as BPA [2], [23]. This immediately raises the question of which hydrogen bond holds 4-α-cumylphenol in the ligand-binding pocket of ERRγ. The X-ray crystal structure of the complex of 4-α-cumylphenol/ERRγ-LBD revealed that the phenol-hydroxyl group of 4-α-cumylphenol makes strong hydrogen bonds with Glu275/Arg316 of ERRγ, whereas the phenyl group undergoes a hydrophobic interaction with the Leu345-isopropyl group [23]. The fact that 4-α-cumylphenol is as potent as BPA was thus substantiated by the similarity of their binding modes in the ERRγ-binding pocket. However, one important structural issue arose; that is, 4-α-cumylphenol did not require the back-and-forth rotation of the Leu345-isobutyl group. In other words, BPA requires the back-and-forth rotation of the Leu345-isobutyl group merely because of the existence of the phenol-B hydroxyl group.

**Figure 4:**
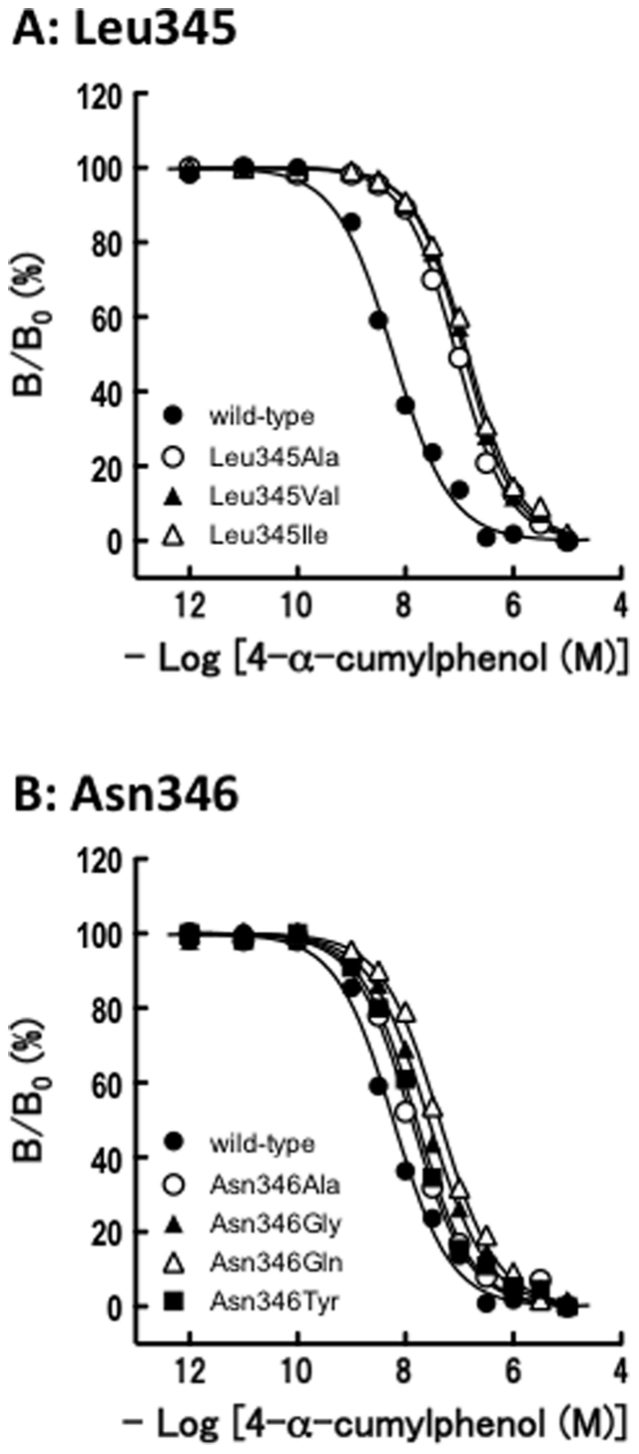
The heterogeneous competitive binding assays between [^3^H]bisphenol A and 4-α-cumylphenol for the wild-type ERRγ-LBD and its mutants. The receptors used were the wild-type ERRγ and its mutant receptors. (**A**) Leu345-substituted ERRγ mutant receptors, and (**B**) Asn346-substituted ERRγ mutant receptors. The graphs show representative dose-dependent binding curves, which give the IC_50_ value closest to the mean IC_50_ from at least three independent experiments.

Given these facts, we evaluated the binding activities of 4-α-cumylphenol for a series of ERRγ mutant receptors. It is clear that all the mutant receptors showed a reduced ability to bind 4-α-cumylphenol just as observed for BPA (Tables 2 and 3). The Asn346 mutant receptors were the only exception, and their activity decrements observed for 4-α-cumylphenol were much smaller (1.7–3.4 times) than those for BPA (4.0–11.3 times) (Table 2). Since 4-α-cumylphenol lacks the BPA's phenol-B hydroxyl group, which is engaged in a hydrogen bond with Asn346, the smaller affinity drops must have been due to the lack of this hydrogen bond (Fig. 4) (Table 2). It should be noted that Leu345 is the most important among the residues for BPA binding, because the drop in affinity was the largest for the Leu345 mutant receptors (Table 2). This implies that Leu345 is responsible for the recognition followed by the strong affinity binding of BPA's phenol-B group.

4-Hydroxytamoxifen (4-OHT) works as an inverse agonist, which suppresses the constitutive high activity of ERRγ [1], [34], [35]. Thus, 4-OHT is a different type of ligand and its binding site appears to be shared only partly with BPA. The molecular volume of 4-OHT is 319.9 Å^3^, while that of BPA is 187.6 Å^3^, indicating that the molecular size of 4-OHT is approximately 1.7-fold larger than that of BPA. When 4-OHT was tested in the competitive binding assays for a series of ERRγ mutant receptors with [^3^H]BPA as a tracer, essentially comparable activity profiles were observed as for BPA (Tables 2 and 3). The results suggested that 4-OHT and BPA share the binding site such as Leu342, Leu345, Asn346, and Ile349 residues on the H7 α-helix.

### Effects of Ala-substitutions on the constitutive biological activity

**Figure 5:**
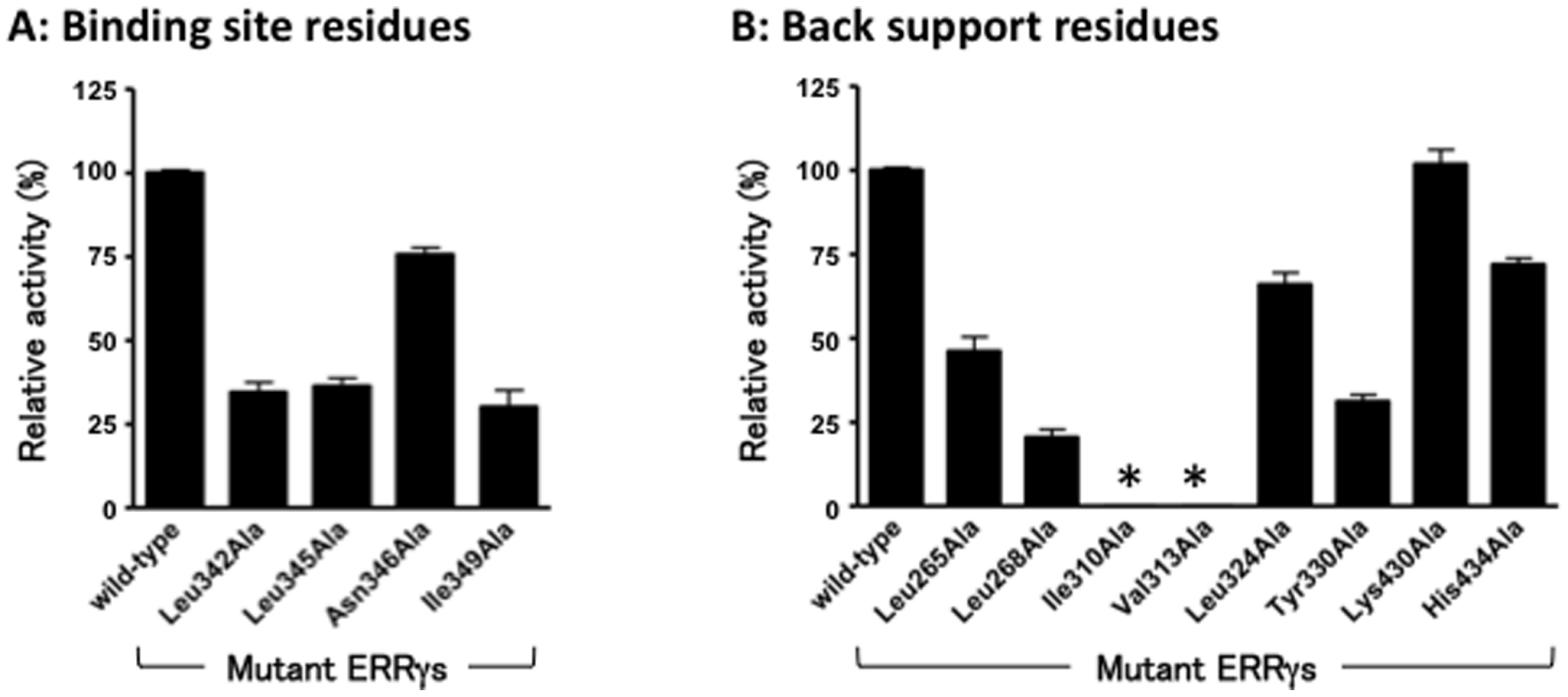
The results of luciferase-reporter gene assay to evaluate the biological activity of ERRγ and its Ala-substituted mutant receptors. The percentage relative potencies of a series of mutant receptors were measured against the basal constitutive activity of the wild-type ERRγ receptor (100%). (**A**) Binding site residues, and (**B**) Back support residues. An internal control that distinguishes the transcriptional level from variations in transfection efficiency was achieved by co-transfecting a second plasmid that constitutively expresses an activity that can be clearly differentiated from SEAP. The mark “*” shows that no basal constitutive activity was observed for Ile310Ala and Val313Ala. The assays were performed in triplicate at least three times (P<0.0001).

ERRγ is a constitutively active self-activating nuclear receptor, and thus ERRγ exhibits almost the full activity without ligand [1], [22], [36]. For a series of Ala-substituted ERRγ mutant receptors, the effects of mutations on such constitutive activity were evaluated by means of the luciferase reporter gene assay, and the results are shown in Fig. 5. When the mutant receptors Leu342Ala-ERRγ, Leu345Ala-ERRγ, Asn346Ala-ERRγ, and Ile349Ala-ERRγ were assayed, Asn346Ala-ERRγ was immediately found to retain approximately 75% of the constitutive activity. In contrast, the other three mutant receptors showed clearly reduced activity: to 30-35% of the activity shown by the wild-type ERRγ (Fig. 5A). These results suggested that a series of amino acids, the pair of Leu342 and Asn346 plus the pair of Leu345 and Ile349, both of which are present in H7, was structurally crucial for the binding of BPA, but the effect of this series on the constitutive activity of ERRγ was rather moderate.

With respect to the constitutive activity, ERRγ must retain its activation conformation, in which α-helix 12 sits on the ligand-binding pocket at an appropriate position. BPA can stay inside the LBP without changing this activation conformation. Most of the amino acid residues inside ERRγ-LBP are simultaneously the binding site of BPA. In addition to Leu342, Leu345, Asn346, and Ile349, the residues Leu268, Leu271, Glu275, Leu309, Arg316, and Tyr326 are also involved. Their Ala-substituted ERRγ mutant receptors were tested previously and the effects of mutations on the constitutive activity were also moderate. Occasionally, however, the Ala-substitutions of back support amino acids were found to cause profound damage to the constitutive activity. For instance, Ile310Ala-ERRγ and Val313Ala-ERRγ were almost completely inactive [24]. This result was also reproduced in the present study as shown in Fig. 5B. Since the conformational damage caused by the Ile310→Ala and Val313→Ala substitutions was found to be irreversible even with a high concentration of BPA, these amino acid residues are crucial for building the activation conformation of ERRγ.

As to other back support amino acids, the Ala-substituted mutant receptors were also tested. The effects of these mutations on the constitutive activity were also moderate, as shown in Fig. 5B. Leu265Ala-ERRγ and Tyr330Ala-ERRγ exhibited approximately 45% and 30% of the constitutive activity of the wild-type ERRγ, respectively, while both Leu324Ala-ERRγ and His434Ala-ERRγ exhibited approximately 70% of the wild-type level of constitutive activity. On the other hand, Lys430Ala-ERRγ showed almost the full constitutive activity of the wild-type ERRγ. All these results clearly indicate that the residual importance of the constitutive activity varies considerably, probably reflecting the difference in structural significance in building of the activation conformation.

## Conclusion

Based on the results from the X-ray crystal structure analysis of the BPA/ERRγ-LBD complex, we carried out an investigation to identify the binding site of the BPA phenol-B ring, by evaluating the receptor binding and biological activities of Ala-substituted mutant receptors. We confirmed that the amino acid residues Leu268, Leu342, Leu345, Asn346, and Ile349 form a receptacle ligand-binding pocket for the BPA phenol-B ring. We also confirmed that a number of amino acid residues, such as Leu265, Leu268, Ile310, Val313, Leu324, Tyr330, Lys430, Ala431 and His434, functioned as back support residues of these binding sites. The present results reveal that the strong binding of BPA to ERRγ is supported by the double-layer binding sites.

## Supporting Information

Figure S1 **SDS-PAGE elution profiles of the ligand-binding domain (LBD) of wild-type ERRγ and a series of mutants.** (**A**) GST-fused ERRγ-LBD, and (**B**) GST-free ERRγ-LBD. Three µg of GST-fused ERRγ-LBD and one µg of GST-free ERRγ-LBD expressed proteins were separated on 12.5% SDS-PAGE gel and stained by Coomassie brilliant blue.
(TIF)

Figure S2 **Temperature-dependent (15–95°C) CD spectra and thermal unfolding curves for representative GST-free ERRγ-LBD proteins.** (**A**) Wild-type ERRγ, (**B**) Leu324Ala ERRγ, and (**C**) Leu342Ala ERRγ. CD spectra in the 200–260 nm UV region are shown by the mean molar ellipticity [*θ*] (degrees⋅cm^2^/dmol). Thermal unfolding curves were depicted, by plotting the mean molar ellipticity [*θ*] at 222 nm. Ala-substitutions of Leu324 (back support residue of Asn346 and Ile349) and Leu342 (direct binding site of BPA's phenol-B group) resulted in inactivity in binding BPA.
(TIF)

Figure S3 **Receptor-binding assays of tritium-labeled bisphenol A (BPA) for the Leu342, Leu345, Asn346, and Ile349 mutant receptors of GST-fused ERRγ-LBD.** (**A**) Saturation binding assays with the curves of total binding (filled circle), non-specific binding (filled square), and specific binding (open circle). (**B**) Scatchard plot analyses showing a single binding mode with a binding affinity constant (*K*_d_) and receptor density (*B*_max_). No Scatchard plot analysis was carried out for the Leu342Ala-ERRγ mutant receptor because of its lack of specific binding in the saturation-binding assay. All the saturation binding assays using [^3^H]BPA were carried out at least three times and a representative result that afforded *K*_d_ and *B*_max_ values close to the means is shown for each mutant receptor.
(TIF)

## References

[1] Takayanagi S, Tokunaga T, Liu X, Okada H, Matsushima A, et al (2006) Endocrine disruptor bisphenol A strongly binds to human estrogen-related receptor gamma (ERRgamma) with high constitutive activity. Toxicol Lett 167: 95–105 10.1016/j.toxlet.2006.08.012.PubMed:17049190 17049190

[2] Okada H, Tokunaga T, Liu X, Takayanagi S, Matsushima A, et al (2008) Direct evidence revealing structural elements essential for the high binding ability of bisphenol A to human estrogen-related receptor γ (ERRγ). Environ Health Perspect 116: 32–38 10.1289/ehp.10587.PubMed:18197296 18197296 PMC2199305

[3] Horard B, Vanacker JM (2003) Estrogen receptor-related receptors: orphan receptors desperately seeking a ligand. J Mol Endocrinol 31: 349–357 10.1677/jme.0.0310349.PubMed:14664699 14664699

[4] Takeda Y, Liu X, Sumiyoshi M, Matsushima A, Shimohigashi M, et al (2009) Placenta expressing the greatest quantity of bisphenol A receptor ERRγ among the human reproductive tissues: Predominant expression of type-1 ERRγ isoform. J Biochem 146: 113–122 10.1093/jb/mvp049.PubMed:19304792 19304792

[5] Kojo H, Tajima K, Fukagawa M, Isogai T, Nishimura S (2006) A novel estrogen receptor-related protein γ splice variant lacking a DNA binding domain exon modulates transcriptional activity of a moderated range of nuclear receptors. J Steroid Biochem Mol Biol 98: 181–192 10.1016/j.jsbmb.2005.10.004.PubMed:16460929 16460929

[6] Giguère V (2008) Transcriptional control of energy homeostasis by the estrogen-related receptors. Endocr Rev 29: 677–696 10.1210/er.20080017.PubMed:18664648 18664618

[7] Poidatz D, Dos Santos E, Brulé A, De Mazancourt P, Dieudonné MN (2012) Estrogen-related receptor gamma modulates energy metabolism target genes in human trophoblast. Placenta 33: 688–695 10.1016/j.placenta.2012.06.002.PubMed:22763271 22763271

[8] Lui K, Huang Y, Choi HL, Yu S, Wong KB, et al (2006) Molecular cloning and functional study of rat estrogen receptor-related receptor gamma in rat prostatic cells. Prostate 66: 1600–1619 10.1002/pros.20429.PubMed:169273302 16927302

[9] National Toxicology Program (NTP) (2008) U.S. Department of Health and Human Services, National Institute of Environmental Health Sciences, National Institutes of Health. Draft NTP brief on bisphenol A Peer Revies. Available on the NTP web site: http://aseh.net/teaching-research/teaching-unit-better-living-through-chemistry/historical-sources/lesson-4-1/National%20Toxicology%20Program-BPADraftBrief-4.14.08.pdf

[10] Viberg H, Lee I (2012) A single exposure to bisphenol A alters the levels of important neuroproteins in adult male and female mice. Neurotoxicolo 33: 1390–1395 10.1016/j.neuro.2012.09.002.PubMed:22981971 22981971

[11] vom Saal FS, Cooke PS, Buchanan DL, Palanza P, Thayer KA, et al (1998) A physiologically based approach to the study of bisphenol A and other estrogenic chemicals on the size of reproductive organs, daily sperm production, and behavior. Toxicol Ind Health 14: 239–260 10.1177/074823379801400115.PubMed:9460178 9460178

[12] Kubo K, Arai O, Omura M, Watanabe R, Ogata R, et al (2003) Low dose effects of bisphenol A on sexual differentiation of the brain and behavior in rats. Neurosci Res 45: 345–356 10.1016/S0168-0102(02)00251-1.PubMed:12631470 12631470

[13] vom Saal FS, Hughes C (2005) An extensive new literature concerning low-dose effects of bisphenol A shows the need for a new risk assessment. Environ Health Perspect 113: 926–933 10.1289/ehp.7713.PubMed:16079060 16079060 PMC1280330

[14] Kamrin MA (2007) The "low dose" hypothesis: validity and implications for human risk. Int J Toxicol 26: 13–23 10.1080/10915810601117968.PubMed:17365142 17365142

[15] Kunz N, Camm EJ, Somm E, Lodygensky G, Darbre S, et al (2011) Developmental and metabolic brain alterations in rats exposed to bisphenol A during gestation and lactation. Int. J Dev Neurosci 29: 37–43 10.1016/j.ijdevneu.2010.09.009.PubMed:20955774 20955774

[16] Yan S, Song W, Chen Y, Hong K, Rubinstein J, et al (2013) Low-dose bisphenol A and estrogen increase ventricular arrhythmias following ischemia-reperfusion in female rat hearts. Food Chem Toxicol 56: 75–80 10.1016/j.fct.2013.02.011.PubMed:23429042 23429042 PMC3637866

[17] Kundakovic M, Gudsnuk K, Franks B, Madrid J, Miller RL, et al. (2013) Sex-specific epigenetic disruption and behavioural changes following low-dose in utero bisphenol A exposure. Proc Natl Acad Sci U S A. doi: 10.1073/pnas.1214056110. PubMed: 23457122. 10.1073/pnas.1214056110 PMC3683772 23716699

[18] Cao J, Rebuli ME, Rogers J, Todd KL, Leyrer SM, et al (2013) Prenatal bisphenol a exposure alters sex-specific estrogen receptor expression in the neonatal rat hypothalamus and amygdala. Toxicol Sci 133: 157–173 10.1093/toxsci/kft035.PubMed:23457122 23457122 PMC3627558

[19] Giguere V (2002) To ERR in the estrogen pathway. Trends Endocrinol Metab 13: 220–225 10.1016/S1043-2760(02)00592-1.PubMed:12185669 12185669

[20] Horard B, Vanacker JM (2003) Estrogen receptor-related receptors: orphan receptors desperately seeking a ligand. J Mol Endocrinol 31: 349–357 10.1677/jme.0.0310349.PubMed:14664699 14664699

[21] Greschik H, Wurtz JM, Sanglier S, Bourguet W, van Dorsselaer, et al (2002) Structural and functional evidence for ligand-independent transcriptional activation by the estrogen-related receptor 3. Mol Cell 9: 303–313 10.1016/S1097-2765(02)00444-6.PubMed:11864604 11864604

[22] Matsushima A, Kakuta Y, Teramoto T, Koshiba T, Liu X, et al (2007) Structural evidence for endocrine disruptor bisphenol A binding to human nuclear receptor ERRγ. J Biochem 142: 517–524 10.1093/jb/mvm158.PubMed:17761695 17761695

[23] Matsushima A, Teramoto T, Okada H, Liu X, Tokunaga T, et al (2008) ERRγ tethers strongly bisphenol A and 4-α-cumylphenol in an induced-fit manner. Biochem Biophys Res Commun 373: 408–413 10.1016/j.bbrc.2008.06.050.PubMed:18582436 18582436

[24] Liu X, Matsushima A, Nakamura M, Costa T, Nose T, et al (2012) Fine spatial assembly for construction of the phenol-binding pocket to capture bisphenol A in the human nuclear receptor estrogen-related receptor γ. J Biochem 151: 403–415 10.1093/jb/mvs008.PubMed:22298789 22298789

[25] Liu X, Matsushima A, Okada H, Tokunaga T, Isozaki K, et al (2007) Receptor binding characteristics of endocrine disruptor bisphenol A for the human nuclear receptor of estrogen-related receptor γ (ERRγ): Chief and corroborative hydrogen bonds of bisphenol A phenol-hydroxyl group with Arg316 and Glu275 residues. FEBS J 274: 6340–6351 10.1111/j.1742-4658.2007.06152.x.PubMed:18005256 18005256

[26] Bradford MM (1976) A rapid and sensitive method for the quantitation of microgram quantities of protein utilizing the principle of protein-dye binding. Anal Biochem 72: 248–254 10.1016/0003-2697(76)90527-3.PubMed:942051 942051

[27] Nakai M, Tabira Y, Asai D, Yakabe Y, Shimyozu T, et al (1999) Binding characteristics of dialkyl phthalates for the estrogen receptor. Biochem Biophys Res Commun 254: 311–314 10.1006/bbrc.1998.9928.PubMed:9918834 9918834

[28] Pichon MF, Milgrom E (1973) Competitive protein binding assay of progesterone without chromatography. Steroids 21: 335–346 10.1016/0039-128X(73)90028-7.PubMed:4693730 4693730

[29] Scatchard G (1948) The attractions of proteins for small molecules and ions. Ann NY Acad Sci 51: 660–672 dio: 10.1111/j.1749-6632.1949.tb27297.x

[30] DeLean A, Munson PJ, Rodbard D (1978) Simultaneous analysis of families of sigmoidal curves: application to bioassay, radioligand assay, and physiological dose-response curves. Am J Physiol 235: E97–E102. PubMed: 686171. 10.1152/ajpendo.1978.235.2.E97 686171

[31] Berger J, Hauber J, Hauber R, Geiger R, Cullen BR (1988) Secreted placental alkaline phosphatase: a powerful new quantitative indicator of gene expression in eukaryotic cells. Gene 66: 1–10 10.1016/0378-1119(88)90219-3.PubMed:3417148 3417148

[32] Sambrook J, Russell DW (2001) Molecular Cloning: A Laboratory Manual, 3rd edn. Cold Springs Harbor Laboratory Press, Cold Springs Harbor, NY.

[33] Dirr H, Reinemer P, Huber R (1994) Refined Crystal Structure of Porcine Class Pi Glutathione S-Transferase (pGST P1-1) at 2.1 Å Resolution. J Mol Biol 243: 72–92 10.1006/jmbi.1994.1631.PubMed:7932743 7932743

[34] Coward P, Lee D, Hull MV, Lehmann JM (2001) 4-Hydroxytamoxifen binds to and deactivates the estrogen-related receptor gamma. Proc Natl Acad Sci USA 98: 8880–8884 10.1073/pnas.151244398.PubMed:11447273 11447273 PMC37529

[35] Wang L, Zuercher WJ, Consler TG, Lambert MH, Miller AB, et al (2006) X-ray crystal structures of the estrogen-related receptor-ligand binding domain in three functional states reveal the molecular basis of small molecule regulation. J Biol Chem 281: 37773–37781 10.1074/jbc.M608410200.PubMed:16990259 16990259

[36] Greschik H, Wurtz JM, Sanglier S, Bourguet W, van Dorsselaer A, et al (2002) Structural and functional evidence for ligand-independent transcriptional activation by the estrogen-related receptor 3. Mol Cell 9: 303–313 10.1016/S1097-2765(02)00444-6.PubMed:11864604 11864604

