# Supplementary figures and images for: A Characteristic Back Support Structure in the Bisphenol A-Binding Pocket in the Human Nuclear Receptor ERRγ
Source: PLoS One. 2014 Jun 30;9(6):e101252. doi: 10.1371/journal.pone.0101252 (PMC4076284; doi:10.1371/journal.pone.0101252)

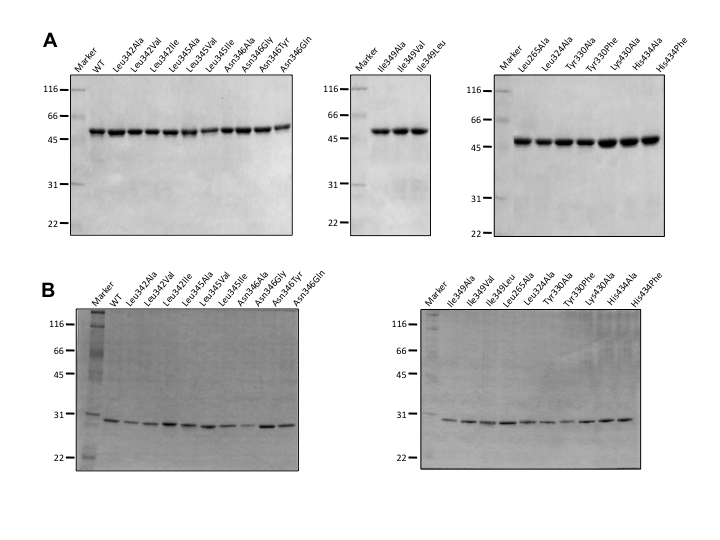

Supplement: Figure S1 — SDS-PAGE elution profiles of the ligand-binding domain (LBD) of wild-type ERRγ and a series of mutants. (A) GST-fused ERRγ-LBD, and (B) GST-free ERRγ-LBD. Three µg of GST-fused ERRγ-LBD and one µg of GST-free ERRγ-LBD expressed proteins were separated on 12.5% SDS-PAGE gel and stained by Coomassie brilliant blue. (TIF) [file pone.0101252.s001.tif]

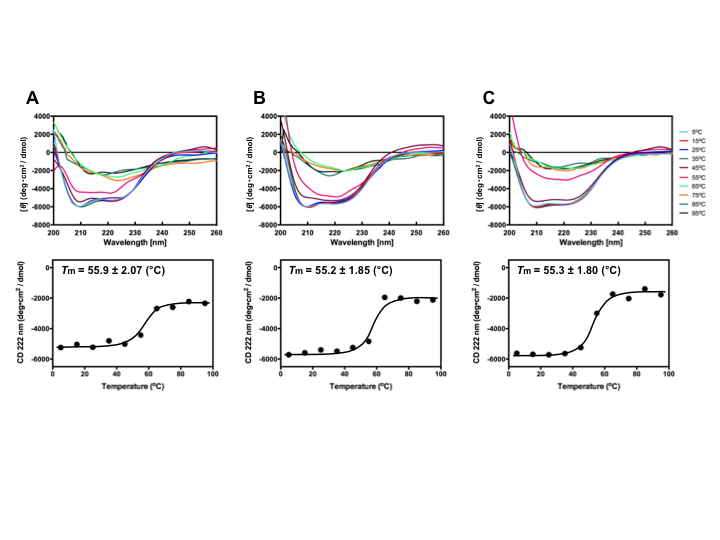

Supplement: Figure S2 — Temperature-dependent (15–95°C) CD spectra and thermal unfolding curves for representative GST-free ERRγ-LBD proteins. (A) Wild-type ERRγ, (B) Leu324Ala ERRγ, and (C) Leu342Ala ERRγ. CD spectra in the 200–260 nm UV region are shown by the mean molar ellipticity [θ] (degrees⋅cm2/dmol). Thermal unfolding curves were depicted, by plotting the mean molar ellipticity [θ] at 222 nm. Ala-substitutions of Leu324 (back support residue of Asn346 and Ile349) and Leu342 (direct binding site of BPA's phenol-B group) resulted in inactivity in binding BPA. (TIF) [file pone.0101252.s002.tif]

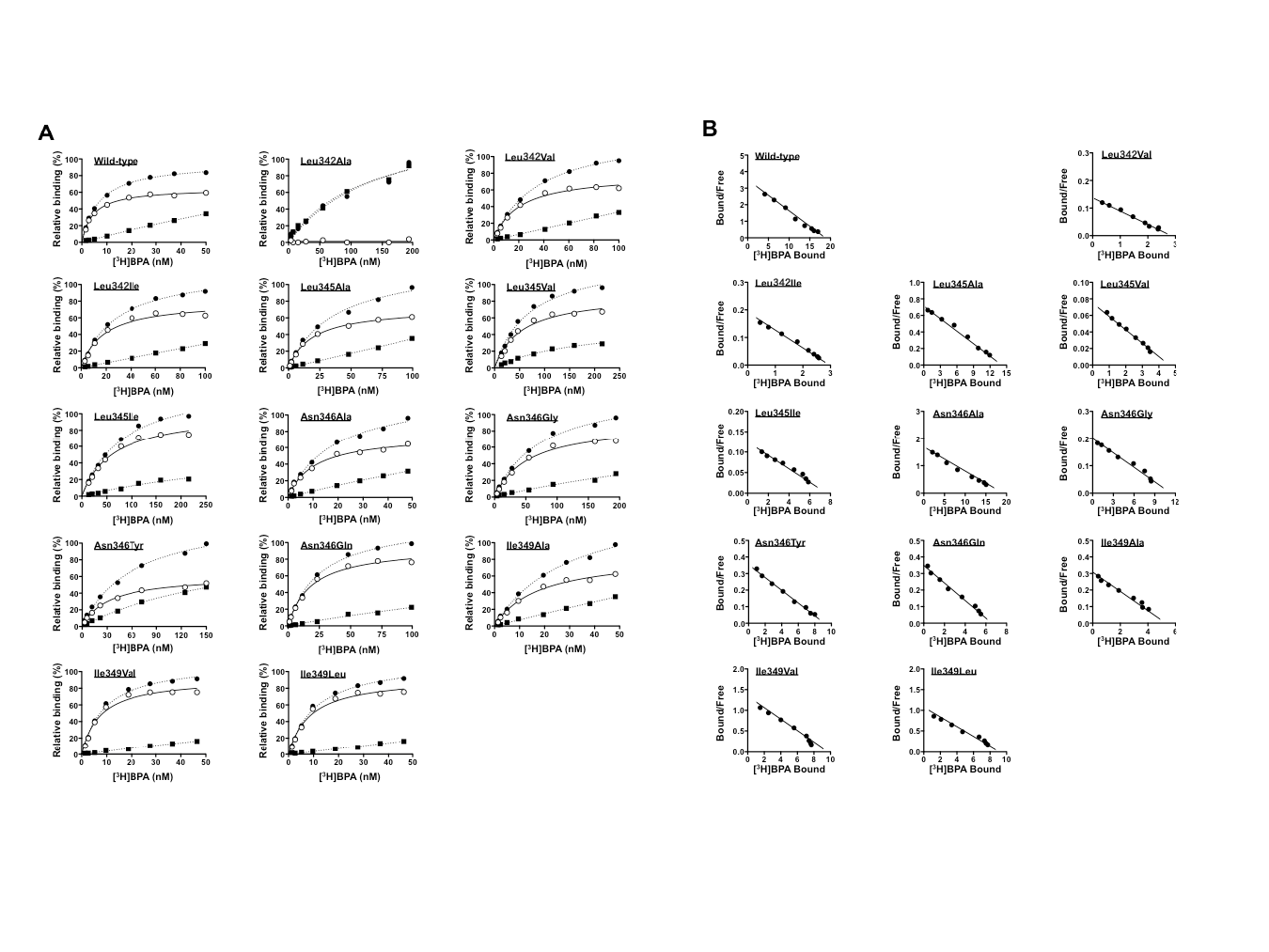

Supplement: Figure S3 — Receptor-binding assays of tritium-labeled bisphenol A (BPA) for the Leu342, Leu345, Asn346, and Ile349 mutant receptors of GST-fused ERRγ-LBD. (A) Saturation binding assays with the curves of total binding (filled circle), non-specific binding (filled square), and specific binding (open circle). (B) Scatchard plot analyses showing a single binding mode with a binding affinity constant (K d) and receptor density (B max). No Scatchard plot analysis was carried out for the Leu342Ala-ERRγ mutant receptor because of its lack of specific binding in the saturation-binding assay. All the saturation binding assays using [3H]BPA were carried out at least three times and a representative result that afforded K d and B max values close to the means is shown for each mutant receptor. (TIF) [file pone.0101252.s003.tif]
